# Transforming Growth Factor-beta Regulation of Ephrin Type-A Receptor 4 Signaling in Breast Cancer Cellular Migration

**DOI:** 10.1038/s41598-017-14549-9

**Published:** 2017-11-03

**Authors:** Ibrahim Y. Hachim, Manuel Villatoro, Lucie Canaff, Mahmood Y. Hachim, Julien Boudreault, Halema Haiub, Suhad Ali, Jean-Jacques Lebrun

**Affiliations:** 1 Department of Medicine, McGill University Health Center, Cancer Research Program, Montreal, QC H4A 3J1 Canada; 2 Sharjah Institute for Medical Research, University of, Sharjah, UAE

**Keywords:** Breast cancer, Metastasis

## Abstract

Breast cancer consists of a range of tumor subtypes with different clinical characteristics, disease prognosis, and treatment-response. Luminal breast cancer has the best prognosis while basal-like breast cancer (BLBC) represents the worst subtype. Transforming growth factor-beta (TGFβ) plays a prominent role in stimulating the migration and invasion of malignant breast cancer cells contributing to tumor progression. In this study, we identified the Ephrin type-A receptor 4 (EPHA4) as a novel target of TGFβ in breast cancer. Moreover, we show that TGFβ induction of EPHA4 gene expression is specific to basal-like tumors and is required for TGFβ-mediated cell migration. We further addressed the mechanism and found EPHA4 to be required for TGFβ-mediated cell migration in breast cancer through TGFβ-induced short term and long term activation of RhoGTPases. Finally, our data revealed a strong association between high EPHA4 expression and advanced tumor stage, aggressive BLBC molecular subtype and poor prognosis. Importantly, we found significant co-expression of EPHA4 and the TGFβ receptor type-2 (TGFβR2) in breast cancer subtypes associated with increased tumor relapse and drug resistance. Together, this study highlight the important role of the TGFβ/EPHA4 signaling axis in mediating tumor aggressiveness and poor patient survival in human breast cancer.

## Introduction

Breast cancer is a heterogeneous disease comprising a wide range of tumor subtypes, each with different clinical characteristics, disease outcome, and response to treatments^1^. Breast tumors can be categorized into immunohistopathological subtypes based on the presence or the absence of estrogen receptor α (ER α), progesterone receptor (PR), and human epidermal receptor 2 (Her2) tyrosine kinase. These subtypes include ER-positive (ER^+^), Her2-positive (Her2^+^), and triple-negative breast cancer (TNBC)^1^. From a clinical standpoint, ER^+^ tumors have the best overall prognosis and are responsive to estrogen-targeted therapies such as the ER-antagonist tamoxifen, aromatase inhibitors, and ER-degrading therapies such as fulvestrant^2,3^. Her2^+^ tumors have an intermediate prognosis and are responsive to trastuzumab (Herceptin)^4,5^. Due to the absence ER and Her2 in TNBC tumors, there are currently no targeted therapies for these patients^5–7^.

The advancement of high-throughput genomic technologies had led to the discovery of breast cancer molecular subtypes that allow the stratification of patients into more precise subgroups with clinical, prognostic and therapeutic values^5^. This molecular classification has also proven useful to predict response to new targeted treatment strategies^5^. Molecular subtypes are based on distinct transcriptional signatures that often correlate with immunohistopathological subtypes but offer more detailed information regarding aggressiveness and therapy responsiveness^8^. Gene expression-profiling of breast tumors defined several subtypes, including luminal A, luminal B, Her2-enriched, basal-like and normal breast-like breast cancer subtypes. In addition, a claudin-low subtype was also characterized with unique biologic features^9^. Each subgroup showed significant differences in the prediction of overall survival, disease-free survival, and risk of relapse^10,11^. Luminal-A tumors are characterized by low proliferation and relapse rates giving them the best prognosis among all other molecular subtypes. In comparison, luminal-B tumors have a higher proliferative index and more aggressive phenotype. Both Her2-enriched and basal-like breast cancer (BLBC) exhibit worse prognosis with BLBC harboring the worst outcome and most reduced survival rates. Moreover, basal like tumors have distinct extra features including loss of differentiation and high proliferation rates^10^.

Not surprisingly, basal-like tumors are closely related to the TNBC subtype, with 70% of all TNBCs being BLBCs^12^. Yet, both terms are not interchangeable and the two groups display significant cellular and genomic heterogeneity when compared to each other^13^. In fact, TNBCs comprise other sub-groups, besides basal-like tumors, including mesenchymal, immunomodulatory, mesenchymal stem–like, luminal androgen receptor subtype and claudin-low tumors^14,15^. In spite of all this heterogeneity, TNBC tumors share common features such as poor prognosis, higher risk of relapse, distant metastasis, enriched cancer stem cell populations^16,17^, resistance to radio- and chemotherapies and lack targeted treatment strategies, further highlighting the need for novel therapies for these breast cancer patients^13^.

Several therapeutic targets are now emerging for the treatment of BLBC and TNBC^18^. Of note, the transforming growth factor β (TGFβ) signaling pathway has been shown to be a potential therapeutic target for BLBC and TNBC^16,17,19–23^. TGFβ is a multifunctional cytokine that is involved in the regulation of numerous fundamental biological processes, including cell proliferation, differentiation, immortalization, and apoptosis, in a context- and cell-specific manner^24–31^. TGFβ is key to the maintenance of homeostasis, acting as a potent tumor suppressor in normal cells and early carcinomas^27–36^. Interestingly, as tumors progress, the tumor-suppressive effects of TGFβ are often lost while TGFβ tumor promoting functions are being favored^31,37^. TGFβ signaling is triggered by _interaction_ of the TGFβ ligand with a complex of two receptors (TGFβRI and TGFβRII), followed by the phosphorylation and activation of the canonical Smad signaling pathway^31^. Activated TGFβRI recruits and phosphorylates the R-Smads, Smad2 and 3, which in turn heterodimerize with the common partner, Smad4. The resulting Smad complex then translocates to the nucleus, where it interacts with various DNA binding partners and transcription factor to regulate the transcription of target genes^24,31^. By evading the TGFβ tumor suppressive effects, while maintaining the pathway functional, cancer cells can take advantage of TGFβ signaling to promote metastasis^31,38^.

Indeed, TGFβ promotes epithelial-mesenchymal transition (EMT), characterized by the loss of epithelial cell junctions, polarity, and cytoskeleton rearrangement, and the acquisition of a mesenchymal gene expression program that allows for cell migration and invasion^26,32,33^. Other mediators of the TGFβ effects on cell migration and invasion involve cell cycle regulators (cyclin D1 and p21)^21,22^, breast cancer anti-estrogen resistance 3 (BCAR3)^20^ and microRNA-584^23^. By repressing the expression of microRNA-584, TGFβ allows for the accumulation of PHACTR1, another regulator of actin dynamics^23^. In addition to Smad pathway, TGFβ also regulates Rho-like GTPases, including RhoA, Rac1 and Cdc42 leading to cell motility, cytoskeletal organization and TGFβ-induced EMT^39,40^. By regulating Rho-like GTPases, TGFβ signaling modulates stress fiber, lamellipodia, and filopodia formation, further leading to cancer cell migration^34–36^.

To better understand and further characterize the molecular mechanisms by which TGFβ promotes cellular migration in BLBC, we performed genomic profiling in BLBC and identified Ephrin type-A receptor 4 (EPHA4), a member of the erythropoietin-producing hepatocellular (Eph) receptor family as a novel target of TGFβ in breast cancer. The Eph receptor family is the largest family of receptor tyrosine kinases (RTKs) in vertebrates and is involved in various cellular processes such as adhesion, migration, proliferation, survival, and differentiation^41^. A previous report also suggested that EPHA4 plays a role in cell-cell interaction-mediated RhoA activation^42^. The Eph family is also implicated in various pathological conditions, predominantly in neurological disease, but also in cancer^43^. Eph receptors and ephrins have been shown to promote cancer progression through crosstalk with oncogenic signaling pathways. In addition, Eph bidirectional signals have been shown to promote tumor angiogenesis^44^.

In this study, we identified EPHA4 as a novel target of TGFβ in BLBC cells. We found EPHA4 to be required for TGFβ-mediated cell migration in BLBC as well as for TGFβ-induced short term and long term activation of several Rho GTPases. We also defined strong correlation between high EPHA4 expression, tumor stage and poor prognosis in basal-like breast cancer. Co-expression of EPHA4 with the TGFβ receptor type-2 also correlated with increased tumor relapse and drug resistance. Together, these results define the TGFβ/EPHA4 signaling axis as a critical regulator of breast cancer cellular migration, tumor aggressiveness and poor patient outcomes and highlight EPHA4 as a potential new prognostic marker for basal-like breast tumors.

## Results

### Eph-ephrin family members are novel targets of TGFβ

**Figure 1:**
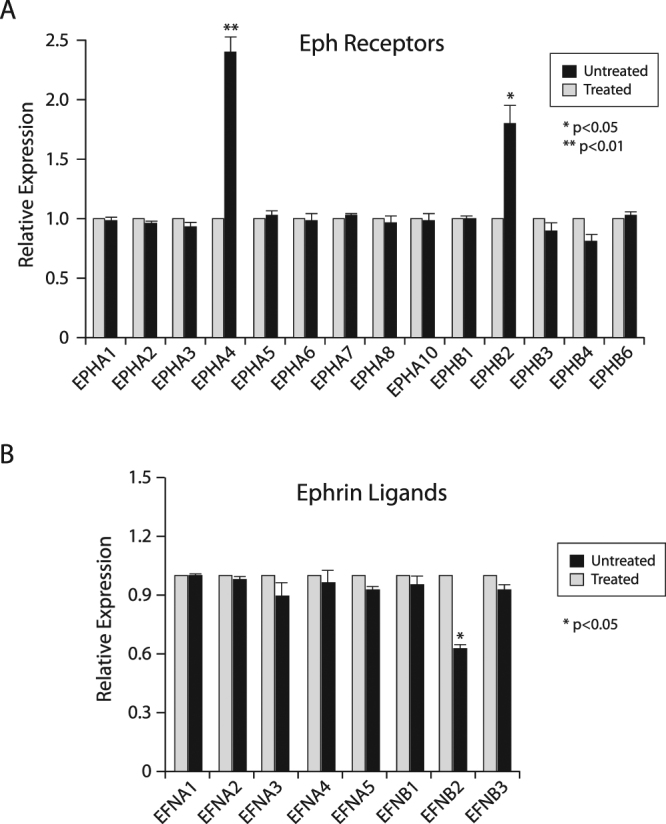
Regulation of Eph Receptors and Ephrin Ligands by TGFβ. Expression of (**A**) Eph receptor and (**B**) ephrin ligand mRNA following 24-hours of TGFβ1 treatment in SCP2 cells relative to untreated cells. Relative expression represents the mean of 3 separate experiments.

Various groups have established that TGFβ has a pro-migratory role in metastatic breast cancer^20–23,31,45–48^. This is particularly evident in BLBC, where TGFβ inhibition has been shown to prevent metastasis^44^. To identify critical mediators of the TGFβ effects on cell migration in BLBCs, we initially performed gene profiling analysis and identified two members of the Eph receptor family – *EPHA4* and *EPHB2* – both of which were up regulated by TGFβ. This was of particular interest as both of these genes have previously been linked to cell migration, invasion, and cancer^43^ and suggested that expression of Eph family members could be regulated by TGFβ. To address this question, we first examined the TGFβ effects on the mRNA levels of the various ephrin receptor (Eph) family members, using QPCR in BLBC cells, using the SCP2 basal-like cell line. As shown in Fig. 1A, we found TGFβ to specifically and significantly upregulate expression of two members of this family, *EPHA4* and to a lesser extent EPHB2. Signaling downstream of the ephrin receptors is highly dependent on their interaction with the ephrin ligands. To then investigate whether ephrin ligands were also regulated by TGFβ we analyzed their expression levels in TNBC cells stimulated or not with TGFβ, Interestingly, as shown in Fig. 1B, TGFβ repressed the expression of *EFNB2*, which codes for ephrinB2, a ligand that binds both EPHA4 and EPHB2^49^. The expression of other ligands from the family was not affected. These results highlight the two ephrin receptors, EPHA4 and EPHB2 and their ligand ephrinB2 as novel TGFβ targets in breast cancer.

### TGFβ differentially regulates the Eph-ephrin system in breast cancer cell lines

**Figure 2:**
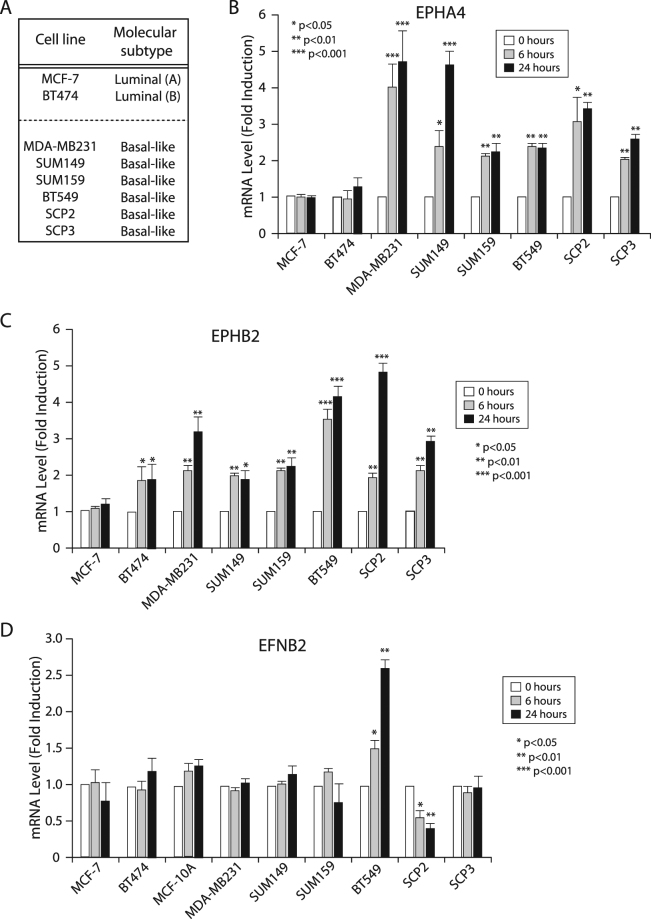
Regulation of EPHA4, EPHB2, and EFNB2 Transcription by TGFβ in a Panel of Human Breast Cancer Cell Lines. Regulation of (**B**) *EPHA4*, (**C**) *EPHB2*, and (**D**) *EFNB2* in (**A**) a panel of luminal and basal breast cancer cell lines following 0, 6, or 24 hours of TGFβ1 treatment. mRNA fold-induction was determined by qRT-PCR and represents a minimum of 3 independent experiments.

Using different cell lines of either luminal or basal origin, we examined the TGFβ effects on EPHA4, EPHB2 and ephrinB2 expression levels. As shown in Fig. 2A,B, TGFβ significantly induced EPHA4 gene expression in all basal types while showed no effect on the two luminal subtypes. Similarly, TGFβ-induced expression of EPHB2 was found in all basal subtypes while not regulated, or to a lower extent, in the luminal cell lines (Fig. 2C). TGFβ-mediated downregulation of the ligand ephrin B2 was confirmed in SCP2 cells but no found in the other basal-like cell lines (Fig. 2D), suggesting that TGFβ primarily regulates expression of the ephrin receptors but nor ligands.

**Figure 3:**
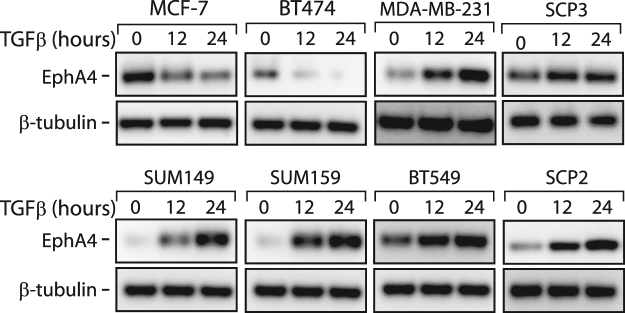
Regulation of EPHA4 Protein Expression by TGFβ in a Panel of Human Breast Cancer Cell Lines. Changes in EPHA4 protein expression in luminal and basal breast cancer cell lines following 0, 12, or 24 hours of TGFβ1 treatment. EPHA4 and β-tubulin protein expression was determined by western blotting.

We then focused our analysis on EPHA4, having it found the most up-regulated target in the receptor group (Figs 1 and 2) and further assessed the TGFβ effect on EPHA4 expression at the protein level. As shown in Fig. 3A, TGFβ strongly induced EPHA4 protein levels in all basal breast cancer cell lines, while decreasing its expression in the two luminal subtypes (MCF7 and BT474). Together, these data indicate that TGFβ significantly induces EPHA4 expression, specifically in BLBC and strongly suggest that EPHA4 acts downstream of TGFβ, to maintain or contribute to the highly metastatic profile in these basal subtype breast cancer cells.

### EPHA4 is Essential for TGFβ-induced Migration

**Figure 4:**
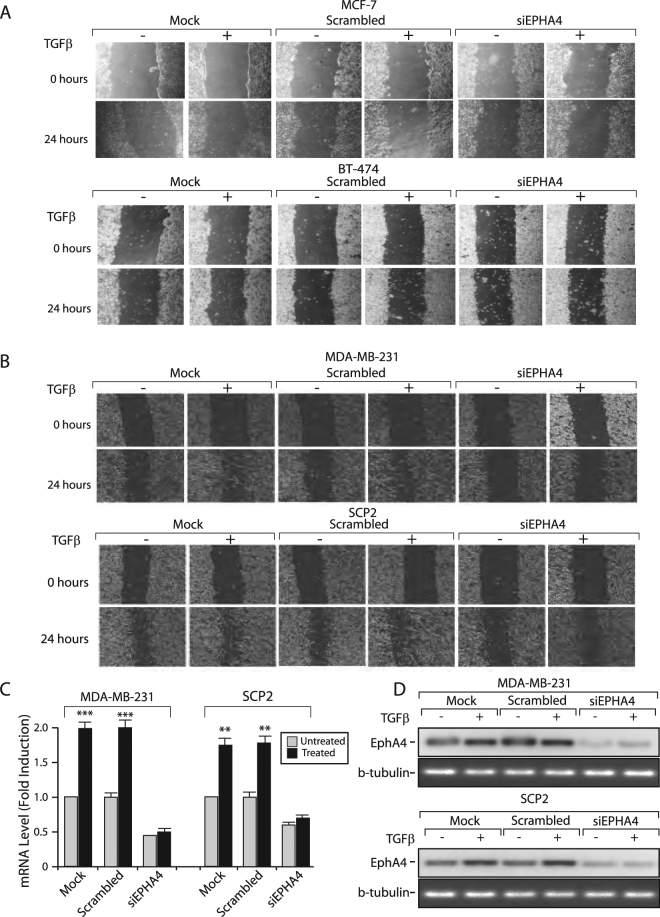
EPHA4 is required for TGFβ-mediated Migration in Human Breast Cancer Cell Lines. **(A)** Luminal MCF-7 and BT-474 and **(B)** Basal-like MDA-MB-231 and SCP2 cell monolayers were transfected with 0 (Mock) or 75 nM of Scrambled or EPHA4 siRNA and seeded to confluence in 6-well plates. Migration was measured following treatment with 0 or 5 ug/mL of TGFβ1 for 24 hours using a wound-healing migration assay. Efficiency of the EPHA4 siRNA knockdown was ensured at both mRNA **(C)** and protein **(D)** levels, using qPCR and Western blotting, respectively.

Given that both TGFβ and Eph family members are involved in the regulation of cell migration, we investigated whether EPHA4 could mediate TGFβ’s pro-migratory effects. As shown in Fig. 4A, TGFβ had no effect on cell migration in the two different luminal cell line tested (MCF7, BT-474). Moreover, knocking-down EPHA4, using a specific siRNA did not altered the migratory potential of the cells. In contrast, our results clearly indicate that TGFβ promotes cell migration in basal-like cells (MDA-MB-231 and SCP2) and that EPHA4 is required for this process (Fig. 4B). Cells were transfected or not with scrambled (control) or a specific EPHA4 siRNA. Migration was assessed following TGFβ treatment for 24 hours using a wound healing migration assay. As shown in Fig. 4B, in basal breast cancer cells, the TGFβ-induced cell migratory effects observed in parental cells and cells transfected with the scrambled siRNA were blocked in cells transfected with the EPHA4 siRNA. Efficiency of the EPHA4 siRNA knockdown in both cell lines was ensured at both mRNA and protein levels, using qPCR and Western blotting, respectively (Fig. 4C,D). Together these results strongly suggest that TGFβ-mediated, EPHA-dependent, cell migration is molecular subtype specific and restricted to basal breast cancer cells.

**Figure 5:**
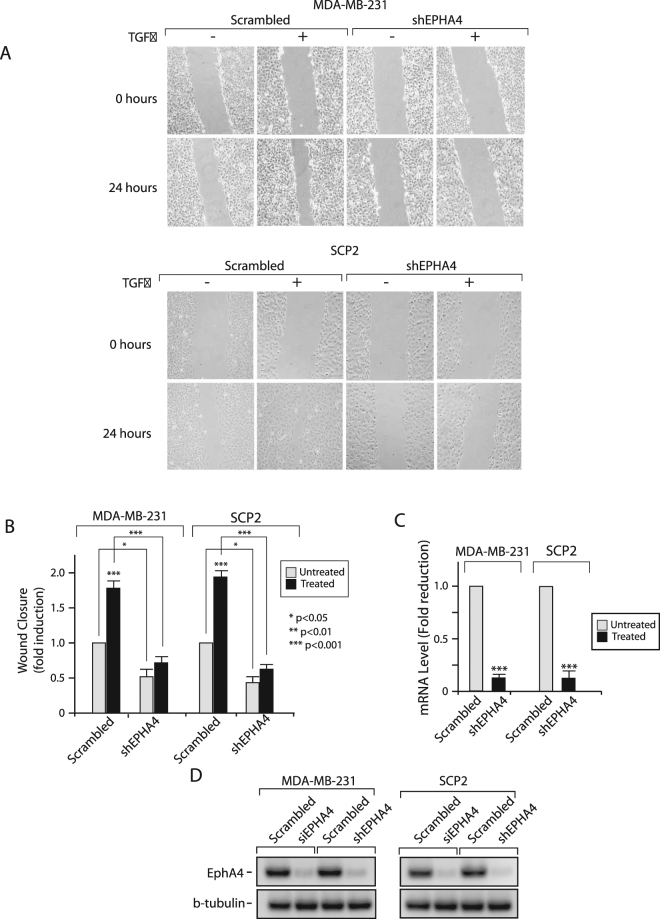
EPHA4 is required for TGFβ-mediated Migration in Human Breast Cancer Cell Lines. **(A)** Stable MDA-MB-231 and SCP2 cell infected with a scrambled or EPHA4 specific shRNA were seeded to confluence in 6-well plates. Migration was measured following treatment with 0 or 5 ug/mL of TGFβ1 for 24 hours using a wound-healing migration assay. (**B**) Wound-closure is graphed relative to the shRNA condition and represents the mean from 3 independent experiments. Pictures are representative of the wound for the indicated conditions at 0 and 24 hours after TGFβ treatment. **(C)** Efficiency of EPHA4 knockdown in MDA-MB-231 and SCP2 cell after shRNA infection. EPHA4 expression was measured by qPCR. **(D)** Efficiency of the EPHA4 knockdown in MDA-MB-231 and SCP2 cells following viral siRNA and shRNA infection. EPHA4 expression was measured by western blot.

To further demonstrate the role and involvement of EPHA4 downstream of TGFβ- mediated cell migration in basal breast cancer, we infected MDA-MB-231 and SCP2 cells with lentiviruses overexpressing scrambled or EPHA4 shRNAs, in order to generate stable cells in which EPHA4 gene expression was specifically knockdown. As shown in Fig. 5A, stable MDA-MB231 and SCP2 cell lines infected with the EPHA4 shRNA showed significant decrease in TGFβ-mediated cell migration, compared to cells infected with the scrambled (control) shRNA. These effects were statistically significant in both cell lines, as indicated in Fig. 5B. Efficiency of the lentiviral infection and EPHA4 shRNA knockdown in both cell lines was ensured at both mRNA and protein levels, using qPCR and Western blotting, respectively (Fig. 5C,D). All together, these results highlight a novel role for EPHA4 in TGFβ-mediated cell migration in basal breast cancer.

### TGFβ Stimulation Results in Activation of Cdc42, RhoA and Rac1

**Figure 6:**
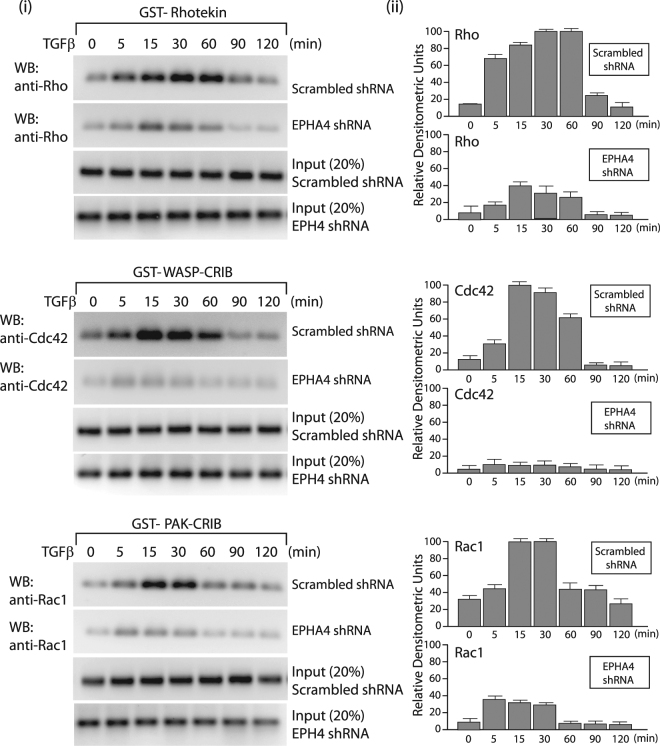
TGFβ Stimulation Results in Activation of Cdc42, RhoA and Rac1 in SCP2 cells. The amount of active, GTP-loaded Rac1, Cdc42, and RhoA was determined by GST pull-down assays with GST-PAK-CRIB, GST-WASP-CRIB, or GST-rhotekin, from TGFβ-stimulated SCP2 stably infected with scrambled or EPHA4 shRNA. Rac1, cdc42, and RhoA were detected by Western blotting by antibodies specific for the respective GTPase (left panels). Relative densitometry units were quantified based on 3 separate experiments (right panels).

The Rho GTPases have been shown to be pivotal regulators of the actin filament system downstream of tyrosine protein kinase receptors^50^. Thus, we next studied whether activation of the TGFβ receptor in basal breast cancer cells could lead to an accumulation of active, GTP-loaded GTPases in control breast cancer cells (scrambled shRNA) versus cells stably overexpressing the EPHA4 shRNA. For these studies, we performed GST pull-down assays with GST-fusion proteins, using the GTPase binding domains of different effectors for these Rho GTPases. Stable MDA-MB-231 and SCP2 cell lines were treated with TGFβ for different times and GST-PAK-CRIB, GST-WASP-CRIB, and GST-rhotekin were used to isolate GTP-loaded Rac1, Cdc42, and RhoA, respectively. The samples were subjected to SDS-PAGE followed by Western blotting with Rac1-, Cdc42-, and RhoA-specific antibodies. Figure 6A shows that GTP-bound Rho, Rac1 and Cdc42 were accumulated 5 min after TGFβ stimulation in control cells (stable lines infected with scrambled shRNA). Activation of the 3 Rho-GTPases by TGFβ is a transient event and rapidly returns to basal levels after 60 mins (upper panels). Interestingly, the TGFβ-mediated effects on RhoA, Cdc42 and Rac1 activation were completely abolished in the absence of EPHA4 (middle panels). These data clearly indicate that TGFβ can activate all 3 RhoGTPases in a time-dependent manner in basal breast cancer cells and that these effects require the presence of EPHA4.

### EPHA4 expression is associated with basal-like breast cancer (BLBC) subtype and its expression is correlated with more advanced tumors and poor clinical outcome

**Figure 7:**
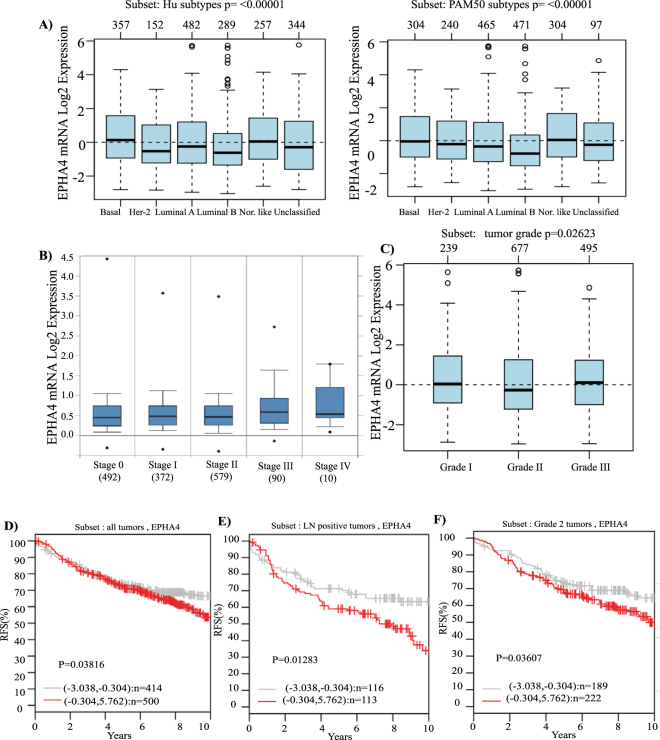
EPHA4 Expression Correlates with Basal-Like Breast Cancer (BLBC) and Poor Relapse-free survival in Breast Cancer Patients. The correlation between high EPHA4 expression and (**A**) Basal-Like Breast Cancer (1881 patients, GOBO database), (**B**) advanced stage (2136 patients,Oncomine database, Curtis dataset), (**C**) high grade (1881 patients, GOBO database) (**D**) relapse-free survival in all patients (GOBO database), **(E**) LN positive tumors (GOBO database), and (**F**) grade II (GOBO database). Kaplan-Meier plots represent median splits into high (red) or low (gray) *EPHA4* expression.

To further evaluate the role of *EPHA4* in breast cancer, next we investigated the association between EPHA4 mRNA levels and tumor grade, stage, molecular subtypes and patient outcome using GOBO and ONCOMINE databases. Interestingly, EPHA4 expression was significantly higher in the highly aggressive basal-like breast cancer subtypes compared to luminal A, luminal B and Her-2 enriched subtypes (P < 0.00001) (Fig. 7A). Moreover, EPHA4 mRNA median levels were also higher in advanced stages, stage III (0.58) and stage IV (0.52) compared to the early stage 0 (0.446), stage I (0.47) and stage II (0.46) (Fig. 7B). Similarly, EPHA4 mRNA levels were significantly higher in poorly differentiated (grade 3) tumors compared to less aggressive (grade 1 and grade 2) tumors (P = 0.02623) (Fig. 7C). These results strongly suggest that high EPHA4 mRNA levels are associated with more advanced tumors and the highly aggressive phenotype. To better evaluate the prognostic value of EPHA4 in breast cancer, we also assessed the association of EPHA4 mRNA levels and patients outcomes using GOBO online database and relapse-free survival (RFS) as an end-point. Indeed, high EPHA4 expression levels showed a significant association with shortened RFS in all patient groups (P = 0.0381), more advanced lymph node (LN) positive groups (P = 0.01283) and grade II tumors (P = 0.03607) (Fig. 7D,E and F).

**Figure 8:**
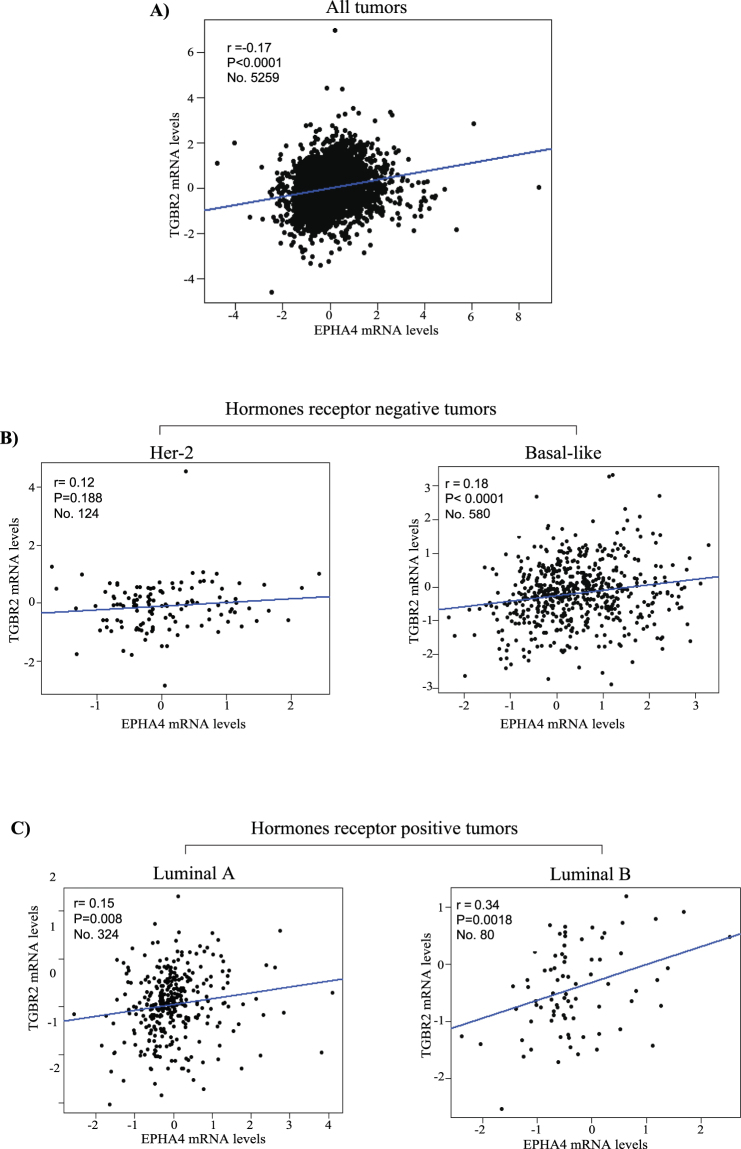
EPHA4 Expression Correlates with TGFBR2 expression in breast cancer patients with stronger association in luminal B and Basal-Like Breast Cancer subtypes in large patients cohorts. The correlation between EPHA4 expression and TGFBR2 expression in all patients group (**A**), hormone receptors negative subtypes (**B**) and hormone receptors positive subtypes (**C**).

Finally, our results also highlight a strong and significant correlation between EPHA4 and TGFBR2 mRNA expression levels (r = 0.17, P < 0.0001) in more than 5000 breast cancer patients samples obtained from Breast Cancer Gene-Expression Miner v4.0 database (Fig. 8A). Moreover, we found significance correlation between EPHA4 and TGBR2 expression in luminal B and basal tumors while no or little association in HER-2 and luminal A tumors (Fig. 8B and C). This association was highest in luminal B (r = 0.34, P = 0.0018) (Fig. 8B) and basal like (Fig. 8C) subtypes (r = 0.18, P < 0.0001), which are both characterized by higher rates of local recurrence, metastasis and drug resistance. Thus, our data revealing higher correlation rates between EPHA4 and TGBR2 in both basal and luminal B tumors (compared to luminal A and HER2) is consistent with the fact these tumors are more aggressive and exhibit a higher rate of metastasis and support a role for TGFβ/EPHA4 in the migratory/invasiveness of breast cancer.

## Discussion

Breast cancer comprises of a range of tumor and cellular subtypes each with different clinical characteristics, disease prognoses, and responses to specific treatments. In order to better predict responses to specific therapies, breast cancer patients are now stratified into 6 molecular subtypes – luminal A, luminal B, Her2-enriched, basal-like, normal-like and claudin-low^5^. Basal-like (BLBC), which are often triple-negative breast cancers (TNBCs), have the lowest prognoses, highest risk of relapse and distant metastasis, and lack targeted-therapies^13^. In breast cancer, TGFβ is understood to be particularly important in promoting the migration and invasion of malignant breast cancer cells. To better characterize the mechanisms through which TGFβ promotes migration in invasive breast cancer cells, we aimed to identify novel pro-migratory gene expression networks downstream of TGFβ and specific to BLBC. In this study, we identified EPHA4 as a novel TGFβ target and highlighted the essential role played by EPHA4 in TGFβ-mediated migration in BLBCs.

EPHA4 is a member of the Eph receptor family, comprising the largest family of receptor tyrosine kinases (RTKs) in vertebrates^49^. Past evidence has revealed a role for EphA2 receptors in breast cancer metastasis, where it can promote cancer cell migration by co-operating with oncogenic pathways, such as Ras-MAPK and Src^51,52^. In other cancer types, the co-operation of EphA2 with PI3K/Akt signaling and EPHA4 with FGFR signaling have similarly been shown to promote migration and invasion^53^. The use of Eph-signaling to potentiate the pro-migratory effects of other pathways supports our results showing that TGFβ increases EPHA4 expression to mediate its pro-migratory effects on breast cancer cells. We also showed that TGFβ strongly increases EPHA4 protein expression in basal cell lines but causes the opposite effect in luminal cells. These data correlate with the essential role played by TGFβ in inducing and promoting tumor metastasis in BLBC but not luminal breast cancer cells^44,49,54^. These indicate that EPHA4 regulate specific functions of TGFβ, such as cell migration in invasive breast cancer cells without affecting the other arm of the TGFβ signaling pathway, such as tumor suppression in luminal and early breast carcinoma. In agreement with these finding, our online database analysis using large cohorts of human breast cancer patients revealed higher EPHA4 mRNA levels in the BLBC compared to other molecular subtypes. This further highlights the important role of EPHA4 in this highly aggressive and more metastatic breast cancer subtype. Thus, new strategies based on targeting EPHA4 in metastatic breast cancer could prove useful and very specific as they will presumably not be interfering with the TGFβ tumor suppressive effects in normal and non-invasive cells.

TGFβ can promote migration through Rho-like GTPases^34,35,55–57^. Our data indicate that the TGFβ-mediated effect on cell migration in BLBC is mediated through the activation of RhoA, Rac1, and Cdc42. These data are consistent with previous reports indicating that EPHA4 could regulated these RhoGTPases during developmental cell migration and differentiation^58,59^ and suggest that the EPHA4/RhoA/Rac1/Cdc42 pathway may represent a major regulator of cellular migration in both normal embryogenic tissue and cancer cells. We found TGFβ-induced activation of Rho-GTPases to occur within 1 hour and to require the presence of EPHA4. We also show that TGFβ-mediated up-regulation of EPHA4 mRNA and protein levels is a dynamic event, reaching a plateau at 6 hrs and 12 hrs, respectively, raising out the possibility that Rho-GTPases activation by TGFβ and TGFβ-mediated increase in EPHA4 expression are independent events. Other reports also implicated some of the RhoGTPases in drug resistance^60^, thus it will be interesting in future studies to further investigate whether TGFβ/EPHA4 signaling could also modulate resistance to chemotherapy treatments in breast cancer patients. Our study also emphasizes the prominent role played by RhoGTPases in cancer progression and suggest that the development of small molecule inhibitors that can block GTPase functions could prove useful as potent anticancer therapeutic targets.

Finally, our results using large datasets from breast cancer patients revealed a significant association between high *EPHA4* mRNA expression levels and poor patient outcome, as illustrated by the shortened relapse free survival in a large cohort of 1881 breast cancer patients. This poor prognostic role was even more significant in the more advanced LN positive tumors, further emphasizing the important pro-migratory role of EPHA4 in advanced invasive breast tumors. Similarly, a recent epidemiological study showed a correlation between high EPHA4 expression and poor OS, as did EphA2, EphA7, and EphB4^50^.

In conclusion, we show that EPHA4 is a novel target of TGFβ specifically up-regulated in BLBC. We also identified an essential role for EPHA4 in mediating the pro-migratory effects of TGFβ in breast cancer cells. Future work will be directed at elucidating the molecular mechanisms through which EPHA4 induces its pro-migratory effects as well as the implications of EPHA4 overexpression *in vivo*.

## Methods

### Cell Culture

MCF-7, MDA-MB-231, SCP2, and SCP3 cell lines were maintained in Dulbecco’s Modified Eagle Medium (DMEM, HyClone Laboratories, Logan, UT, USA) supplemented with 10% fetal bovine serum (FBS, Wisent, St-Brono, Qc, Canada), 2 mM L-glutamine (HyClone), and 100 units/mL of penicillin/streptomycin (HyClone). For experiments, these cell lines were grown to 80% confluency and serum-starved in DMEM supplemented with 1% FBS and 2 mM L-glutamine overnight. SUM149 and SUM159 cell lines were maintained in F-12 Ham’s Nutrient Mixture (HyClone) with 5% FBS, 5 ug/mL insulin (Sigma-Aldrich, St. Louis, MO, USA), 10 ng/mL epidermal growth factor (EGF, Sigma), 1 ug/mL hydrocortisone (Sigma), and 100 units/mL of penicillin/streptomycin. For experiments, these cell lines were grown to 80% confluency and serum-starved by culturing in F-12 Ham’s Nutrient Mixture overnight. HEK293T cells were maintained in DMEM (HyClone) supplemented with 10% FBS (Wisent), 2 mM L-Glutamine (HyClone) but no antibiotics. All cell lines were cultured at 37 °C, 5% Co_2_, culture medium was changed every 2–3 days, and cells were passaged once monolayers reached approximately 95% confluence.

### Transfection

Cell lines were cultured to approximately 80% confluence in 6-well plates. Monolayers were transfected with 50 nM of scrambled or EPHA4 siRNA (Sigma) using Lipofectamine™ 2000 (Invitrogen) according to the manufacturer’s protocol. The following morning (approximately 16 hours after transfection), the transfection medium was removed and replaced with culture medium as indicated above. Within 24 hours, monolayers were then serum-starved and treated with TGFβ. Generation of stable lentivirus cell lines - For stable cell line generation, HEK293T cells were grown to 90% confluency in 10 cm dishes. Transfer vectors (scrambled or EPHA4 shRNAs (SHCLNG-NM_004438, Sigma)), pMD2.G (envelope plasmid) and psPAX2 (packaging plasmid) were transfected with polyethylenimine hydrochloride (bPEI, Sigma). After 25 minutes of incubation at room temperature, transfection complexes were added dropwise to HEK293T cells. Cells were then incubated for 12–16 hours at 37 °C in 5% CO2. Next day, the medium was changed with an induction medium (DMEM supplemented with 4.5 g/l D-glucose, 4 mM L-glutamine and 1 mM sodium pyruvate) with 10% FBS, 1% Penicillin/Streptomycin mix, 25 mM HEPES and 3 mM caffeine. The viral titer was checked by Lenti-X GoStix at 48 h after transfection and stored at −80 °C until infection.

SCP2 and MDA-MB-231cells were infected with packaged scrambled or EPHA4 shRNAs and pools of stable cells were selected with 10 ng/ml of puromycin (Invitrogen).

### TGFβ Treatment of Cells

Cell monolayers were serum-starved as indicated above and treated with 5 μg/mL of TGFβ1 (PeproTech, Rocky Hill, NJ, USA) for the indicated time points.

### Polymerase Chain Reaction (PCR)

Total RNA was extracted using Trizol reagent (Invitrogen) according to the manufacturer’s protocol and reverse transcription was carried out using random hexamers and M-MLV Reverse Transcriptase (Invitrogen) as per manufacturer’s protocol. Subsequently, real time qPCR was performed using SsoFast^TM^ EvaGreen Supermix® (BioRad) in a RotorGene™ 6000 PCR detection system (Corbett Life Science) and corresponding software according to the manufacturer’s protocol. Conditions for qPCR were as follows: 95 °C for 30 sec, 40 cycles of 95 °C for 5 sec and 60 °C for 20 sec. Primer sequences are listed in Table 1.

**Table 1:** Primers sequences.

Target name	Sequence (5′–>3′)	Amplicon size (bp)
EPHA1	Forward 5′-ACAGGGAGCCCTGGACTTTA-3′ Reverse 5′-CCTCAGGGTCCCTCGATACA-3′	106
EPHA2	Forward 5′-ACTGCCAGTGTCAGCATCAA-3′ Reverse 5′-CCTCGTACTTCCACACTCGG-3′	127
EPHA3	Forward 5′-CAAGACAGTTTGCTGCGGTC-3′ Reverse 5′-TGCTTTTCATAGTATTTGACCTCGT-3′	169
EPHA4	Forward 5′-AGTGATGTCGTACGGGGAGA-3′ Reverse 5′-ACAAGGCAGTGTTAGGTCTGG-3′	260
EPHA5	Forward 5′-GACTCCCGAGTCTCCCTTCG-3′ Reverse 5′-CCATGACAGTGCGTGAATCC-3′	300
EPHA6	Forward 5′-ACTATGAGAAAGAACATGAGCAGC-3′ Reverse 5′-GGAGAGTGAATCCGCCAACA-3′	246
EPHA7	Forward 5′-TTCCAGGCACCAAAACCTACA-3′ Reverse 5′-TCAAACTGCCCCATGATGCT-3′	265
EPHA8	Forward 5′-CTCACGTATCCGGCTCATGG-3′ Reverse 5′-TCGCGCAGGGTAAACTTGAT-3	182
EPHA10	Forward 5′-GACCGCCGAGGAAGTTATCC-3′ Reverse 5′-ATGGGACGGTCGTGTTCATC-3′	120
EPHB1	Forward 5′-AAGGATACCGAGAAGCCACC-3′ Reverse 5′-GTAGGTGCGGATGGTGTTCA-3′	283
EPHB2	Forward 5′-GTGTGTAACAGACGGGGGTT-3′ Reverse 5′-CCCGACTTGAGCGTCTTGAT-3′	284
EPHB3	Forward 5′-CCAACGGAGTCATCCTGGAC-3′ Reverse 5′-CGAAGACAAGCCCAGCTGTA-3′	276
EPHB4	Forward 5′-GGGCTACGTCCTGACTTCAC-3′ Reverse 5′-GACCTCGTAGTCCAGCACAG-3′	225
EPHB6	Forward 5′-GTTAGGGAACAGAGTGGCGG-3′ Reverse 5′-CAGATGTCTCTCCGGTGGTG-3′	110
EFNA1	Forward 5′-GCTACTACTACATCTCTCACAGTCC-3′ Reverse 5′-TGCTATGTAGAACCCGCACC-3′	96
EFNA2	Forward 5′-CTACATCTCTGCCACGCCTC-3′ Reverse 5′-CGGGCTGCTACACGAGTTAT-3′	136
EFNA3	Forward 5′-GCCTTCTCTCTGGGCTACG-3′ Reverse 5′-GCAGACGAACACCTTCATCCT-3′	108
EFNA4	Forward 5′-ACCCTTCTCCCTCGGCTTTG-3′ Reverse 5′-CACCCTGATGTGCCACTCTCT-3′	177
EFNA5	Forward 5′-AGGACTCCGTCCCAGAAGAT-3′ Reverse 5′-TGGGATTGCAGAGGAGATGT-3′	236
EFNB1	Forward 5′-AGGCCAGAGCAGGAAATACG-3′ Reverse 5′-GCATTGGGATCTTGCCCAAC-3′	203
EFNB2	Forward 5′-TGGACAAGATGCAAGTTCTGCT-3′ Reverse 5′-AACCGAGGATGTTGTTCCCC-3′	176
EFNB3	Forward 5′-CCTGGAGCCTGTCTACTGGA-3′ Reverse 5′-TGGGCGATCACAAGTGAGAA-3′	235
GAPDH	Forward 5′-GCCTCAAGATCATCAGCAATGCCT-3′ Reverse 5′-TGTGGTCATGAGTCCTTCCACGAT-3′	104

### Western Blot Analysis

Following treatment, monolayers were placed on ice and washed with cold PBS. Cells were lysed using protein lysis buffer (10 mM Tris-HCl pH 7.5, 150 mM NaCl, 5 mM EDTA, 30 mM Na_4_P_2_O_7_, 50 mM NaF, 1 mM Na_3_VO_4_, 1% Triton X-100, 1 mM PMSF, 10 ug/mL leupeptin, 10 ug/mL aprotinin, and 10 ug/mL pepstatin). Lysates were collected using cell scrapers, transferred to microcentrifuge tubes, and centrifuged at 14000 rpm for 15 minutes at 4 °C. Protein concentration was quantified using the Pierce BCA Protein Assay Kit (Thermo Scientific) according to the manufacturer’s protocol. Supernatant was combined with 6X SDS-buffer, heated at 65 °C for 5 minutes, and stored at −20 °C.

Samples were resolved on SDS-polyacrylamide gels, transferred to a nitrocellulose membrane, and blocked for 1 hour in 5% milk in TBST. Membranes were incubated with primary antibody diluted (1:1000) in antibody buffer overnight. The following day, membranes were washed and incubated with secondary antibody diluted (1:10000) in 5% milk in TBST for 1 hour at room temperature. Membranes were then developed using ECL, film, and radiography.

Primary and secondary antibodies were all from Santa Cruz Biotechnology: EPHA4 (sc-921), Cdc42 (sc-8401), Rac (sc-11), RhoA (sc-179) and β-tubulin (sc-2005). Secondary antibodies were goat anti-rabbit IgG-HRP (sc-2030) and goat anti-mouse IgG-HRP (sc-2031).

### Wound-Healing Migration Assay

Cells were grown to confluence in 6-well plates, transfected, and serum-starved as indicated above. A scratch (wound) was performed in the centre of the monolayer using a sterile 20–200 μL pipette tip. The monolayer was then washed with PBS to remove any debris before taking pictures of the wound (zero-time point). Cells were then treated with TGFβ as indicated above and pictures of the wound were taken after 24 hours using bright field light microscopy with a Nikon Eclipse E600 microscope and a RS Photometrics CoolSNAP camera. Images were analyzed using Cell Profiler Cell Image Analysis Software (33). Migration was calculated for each condition by subtracting the wound area after 24 hours from the wound area at 0 hours. Fold-induction of migration was calculated by dividing the migration for each condition by the migration in the non-transfected and non-treated condition.

### GST-tagged protein production in E. coli

Glutathione-*S*-transferase (GST)-fusion proteins (GST-WASP-CRIB, GST-PAK-CRIB, and GST-rhotekin). GST-p21-activated kinase (PAK)-Cdc42/Rac interactive binding (CRIB) is a fragment encoding amino acid residues 56–267 of human PAK1B. GST-Wiskott-Aldrich Syndrome protein (WASP)-CRIB is encoding amino acid residues 201–321. GST-rhotekin encodes a fragment which encompasses the Rho-binding domain (amino acid residues 1–89) of mouse rhotekin. *E. coli* BL21(DE3) (Invitrogen) expressing GST-fusion proteins were cultured in LB containing 100 µg/ml ampicillin with shaking overnight at 37 °C. The cultures were diluted 1/1000 into 500 ml of 2x YT medium (100 µg/ml ampicillin) and grown with vigorous agitation at 37 °C to an OD600 of 0.5. Isopropyl-1- thio-β-D-galactopyranoside (IPTG) (Invitrogen) was added to a final concentration of 1 mM and incubation continued at 25 °C for 8 hours. Bacteria were pelleted by centrifugation at 4 °C, resuspended and lysed in PBS buffer with protease inhibitors (Sigma) and 1% Triton X-100, followed by sonication on ice. Cell debris were pelleted by centrifugation and supernatants were stored at −80 °C. GST fusion proteins were batch purified with Glutathione Sepharose 4B beads (GE Healthcare) by rocking at 4 °C overnight. The beads were pelleted by centrifugation at 1000 × g at 4 °C and washed 5 times with cold PBS and resuspended in binding buffer (150 mM KCl, 20 mM HEPES, 0.1% Nonidet P-40, 5 mM MgCl2, 10% glycerol, 1 mM DTT, 0.5 mM EDTA). The purity of the GST-fusion proteins was assessed by SDS-PAGE.

### GST Pull-Down Assays

SCP2 cells were stimulated with TGF-β1 as indicated in the figure legends. The cells were washed with ice-cold PBS. Ice-cold lysis buffer (50 mM Tris-HCl pH 7.5, 1% Triton X-100, 0.5% sodium deoxycholate, 0.1% SDS, 500 mM NaCl, 10 mM MgCl_2_, 10 μg/ml aprotinin, and 1 mM PMSF) was added. Lysates were centrifuged at 14,000 rpm for 10 min at 4 °C. The supernatants were incubated overnight at 4 °C on a rotating shaker with GST-PAK-CRIB, GST-WASP-CRIB, or GST-rhotekin for detection of activated Rac1, Cdc42, and RhoA, respectively. The beads were then pelleted by centrifugation at 1000 × g (4 °C) and washed 4 times with cold binding buffer. The beads were boiled in Laemmli buffer and the released proteins were separated by SDS-PAGE and analyzed by western blot.

### Data Mining

GOBO Gene Set Analysis Tumors module was used to evaluate the association between EPHA4 mRNA levels and molecular subtypes in 1881 breast cancer patients. For better accuracy, two common molecular subtypes’ classification methods were used in the study. Hu^61^ is based on a 306 gene signature that can distinguish different breast cancer subtypes with distinct patient outcome, while PAM 50^12^ relies on a 50 gene signature. The same database was used to evaluate the association between EPHA4 and patient outcome. Moreover, ONCOMINE data-mining platform, was used to investigate the association between EPHA4 mRNA levels and tumor stage. In addition, the Breast Cancer Gene-Expression Miner v4.0 database was used to evaluate the correlation between EPHA4 and TGFBR2 mRNA expression levels.

### Statistical Analysis

Results are expressed as mean ± standard deviation of at least 3 independent experiments. Unless mentioned, statistical differences were determined by two-tailed unpaired *t-*test. *p* < 0.05 was considered statistically significant. In addition, ANOVA test was used to evaluate the statistical significance of EPHA4 expression in the different molecular subtypes. To evaluate the correlation between EPHA4 and TGFBR2 in clinical breast cancer samples corresponding to the different molecular subtypes, Gene expression correlation tool of the breast cancer gene-expression miner v4.0 was used. The strength of the correlation was evaluated using the Pearson correlation coefficient (r). All experimental protocols and procedures were performed in accordance to McGill University regulations. All experimental protocols and procedures were approved by McGill University.
