# Author Correction: Transforming Growth Factor-beta Regulation of Ephrin Type-A Receptor 4 Signaling in Breast Cancer Cellular Migration

**DOI:** 10.1038/s41598-022-24542-6

**Published:** 2022-12-14

**Authors:** Ibrahim Y. Hachim, Manuel Villatoro, Lucie Canaff, Mahmood Y. Hachim, Julien Boudreault, Halema Haiub, Suhad Ali, Jean-Jacques Lebrun

**Affiliations:** 1 Department of Medicine, McGill University Health Center, Cancer Research Program, Montreal, QC H4A 3J1 Canada; 2 Sharjah Institute for Medical Research, University of Sharjah, Sharjah, UAE

Correction to: *Scientific Reports* https://doi.org/10.1038/s41598-017-14549-9, published online 03 November 2017

This article contains an error in Figure 5.

As a result of an error in the figure assembly, the representative image provided for SCP2 cells infected with EPHA4-specific shRNA, in the TGF-β-negative condition displayed in panel A of Figure 5 is incorrect. The corrected Figure 5 and its accompanying legend appear below.

**Figure 5:**
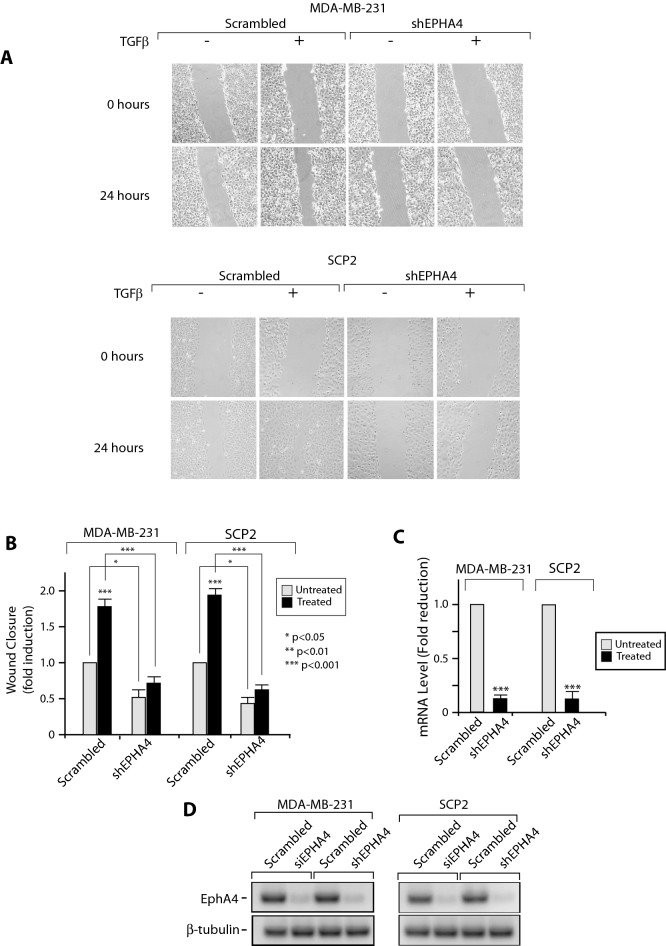
EPHA4 is required for TGFβ-mediated migration in human breast cancer cell lines. Stable MDA-MB-231 and SCP2 cell infected with a scrambled or EPHA4 specific shRNA were seeded to confluence in 6-well plates. Migration was measured following treatment with 0 or 5 ug/mL of TGFβ1 for 24 hours using a wound-healing migration assay.

These changes do not affect the conclusions of the Article.

